# Cost utility analysis of HIV pre exposure prophylaxis among men who have sex with men in Israel

**DOI:** 10.1186/s12889-020-8334-4

**Published:** 2020-02-27

**Authors:** G. M. Ginsberg, D. Chemtob

**Affiliations:** 1 Health Economics Consultant, Derech Hebron 79/3, 9339006 Jerusalem, Israel; 2 Braun School of Public Health and Community Medicine, Faculty of Medicine, Hebrew University-Hadassah, Jerusalem, Israel; 3 Department of Tuberculosis and AIDS, Ministry of Health, Jerusalem, Israel

**Keywords:** HIV infection, Prevention, PrEP, Cost-utility analysis, AIDS, Israel

## Abstract

**Background:**

Between 2011 and 2015, Men who have sex with men (MSM) accounted for nearly half of new HIV cases among men in Israel. This study carries out a cost-utility analysis of PrEP (HIV Pre Exposure Prophylaxis), an antiretroviral medication that can protect against the acquisition of HIV infection, whose incidence rate in Israel is around 1.74 per 1000 MSM.

**Method:**

Epidemiological, demographic, health service utilisation and economic data were integrated into a spread-sheet model in order to calculate the cost per averted disability-adjusted life year (DALY) of the intervention from a societal perspective, in mid-2018 US$ using a 3% discount rate. Cost utility analyses were performed for both types of PrEP delivery (continuous regimen and on-demand), together with sensitivity analyses on numbers of condom users who take up PrEP (baseline 25%) and subsequently abandon condom use (baseline 75%), PrEP efficacy (baseline 86%), PrEP prices and monitoring costs.

**Results:**

Around 21.3% of MSM are high risk (as defined by having unprotected anal intercourse). Offering PrEP to this group would have a ten year net cost of around 1563 million USD, preventing 493 persons from becoming HIV-positive, averting around 1616 DALYs at a cost per averted DALY of around 967,744 USD. This will render the intervention to be not cost-effective. PrEP drug prices would have to fall dramatically (by 90.7%) for the intervention to become cost-effective (i.e. having a cost per averted DALY less than thrice GNP per capita) in Israel. PrEP remains not cost-effective (at 475,673 USD per averted DALY) even if intervention costs were reduced by using an “on demand” instead of a daily schedule. Even if there were no changes in condom use, the resultant 411,694 USD cost-utility ratio is still not cost-effective.

**Conclusions:**

Despite PrEPs high effectiveness against HIV, PrEP was found not to be cost-effective in the Israeli context because of a combination of relatively low HIV incidence, high PrEP costs, with a likelyhood that some low-risk MSM (ie: who use condoms) may well begin taking PrEP and as a consequence many of these will abandon condom use. Therefore, ways of minimizing these last two phenomena need to be found.

## Background

Between 2011 and 2015, men who have sex with men (MSM) accounted for over 30% of all new HIV infections and 46% of new HIV cases among men notified to the Israeli Ministry of Health [1]. Regular use of an intervention referred to as PrEP (HIV Pre Exposure Prophylaxis), which consists of antiretroviral medication (currently *tenofovir* with or without *emtricitabine [TDF/FDC]),* by uninfected persons can protect against the acquisition of HIV infection.

The efficacy of oral PrEP against HIV has been demonstrated in four randomized controlled trials [2–5]. In 2015, the World Health Organization (WHO) recommended that PrEP should be offered as an additional prevention option for people at substantial risk (> 3 per 100 person years) of HIV infection as part of a combination prevention approach [6].

In June 2017, PrEP was included in the official drug registry of Israel, and clinical guidelines were developed [7]. Despite having incidence rates, in MSM who exhibit high risk behaviour (around 0.47 per 1000), far below the WHO guidelines, Israeli Health Maintenance Organizations (HMOs) began to offer PrEP to their members who pay a voluntary premium beyond the standard national insurance coverage afforded to all citizens (77% of Israelis pay for premium coverage.).

Physicians, certified in PrEP prescription and management, dispensed PrEP to eligible persons. The set copayment is between 84 and 103 USD for a monthly supply of PrEP.

This paper aims to investigate as to whether or not the provision of PrEP is cost-effective, by carrying out a cost utility study of PrEP use by MSM in Israel, based on modelling data. This will provide the basis of economic-epidemiological evidence to aid the Israeli Basket of Health Services committee in their decision as to whether or not to include PrEP for MSM into the above national basket on a long-term basis, as opposed to the current situation wherein PrEP is exclusively available for premium-paying HMO members.

## Methods

Data were obtained from the Israeli Central Bureau of Statistics (Demographic, Mortality, Employment Costs), Israeli Ministry of Health (Cost Data), Israeli Ministry of Health Department of Tuberculosis and AIDS (HIV Care Protocols, Prevalence and Incidence), WHO/CDC (Utility Weights). The above data were supplemented by data from a search of the world literature from 1990 to mid-2018 on PubMED, using keywords: (HIV or AIDS) and (prevention or prophylaxis or PrEP), supplemented by unpublished conference proceedings (PrEP Effectiveness, Side-Effects, transitions from HIV to AIDS and from AIDS to death).

Age-specific data for HIV-positive MSM (ie: infected) persons for 2015 were obtained from the National HIV/AIDS registry maintained by the Department of Tuberculosis and AIDS of the Ministry of Health. The age-specific distribution for HIV-negative MSM was based on an estimated 78,013 MSM in Israel, representing 3% of the male population [8] and an adjustment for these “susceptibles” being one year younger than HIV-positive persons on average [9].

### Cost-utility analysis

#### Model development

An Excel-based spread-sheet model was constructed, incorporating intervention costs, treatment costs, disability weights, epidemiology and health service utilization, mortality rates, PrEP efficacy, and indirect costs. (Parameter values listed in Appendix 1).

#### Cost utility calculation

The model calculated the effect of PrEP on the incidence and mortality from HIV/AIDS in Israel in the MSM risk group as a basis of the calculation of the gold-standard “cost-utility ratio” (CUR) to estimate the cost-effectiveness of providing for ten years:-

- a continuous PrEP regimen.
- a PrEP regimen “on demand” countries, such as that used in France [10].

The cost utility ratio (CUR) calculated the net costs per averted Disability Adjusted Life Year (DALY) added as a result of using PrEP, using the standard formula:

$$ \mathrm{Net}\ \mathrm{Costs}\ \mathrm{per}\ \mathrm{a}\mathrm{verted}\ \mathrm{DALY}=\frac{\left(\mathrm{Costs}\ \mathrm{of}\ \mathrm{PrEP}-\mathrm{Savings}\ \mathrm{in}\ \mathrm{treating}\ \mathrm{HIV}\&\mathrm{AIDS}\right)}{\mathrm{DALYs}\ \mathrm{averted}\ \mathrm{a}\mathrm{s}\ \mathrm{a}\ \mathrm{result}\ \mathrm{of}\ \mathrm{decreased}\ \mathrm{mortality}\&\mathrm{morbidity}} $$

Costs were viewed from a societal perspective at mid-2018 price levels and therefore included costs not only incurred by the health and welfare services but also included work absences, transport costs to receive treatment, and premature burial costs. All future costs and averted DALYs were discounted at an annual rate of 3%.

We valued the societal costs of premature mortality by using a methodology that calculated discounted premature burial costs alongside a “zero friction cost” [11]. Zero friction costs assume that a worker, upon death, will be replaced by another unemployed worker with a similar skill set. We refrained from using discounted future productivity losses (whether or not adjusted by discounted future consumption losses) as these do not represent real resource costs, even though they are invariably used by advocates of disease specific interventions for advocacy purposes to inflate the monetary impact of their disease.

### Intervention costs

Intervention costs were based on PrEP costs according to a daily regimen plus the costs of the treatment protocols recommended by the Ministry of Health, which consisted of visits to the prescribing physician as well as numerous laboratory tests. Co-payment costs were excluded from the analysis as these are basically a transfer payment from the individual to the pharmaceutical company via the individuals’ HMO.

A sensitivity analysis was performed using unit costs of monitoring tests provided by one of the HMOs for the research evaluation study, since these more closely represent the true resource costs as opposed to the Ministry of Healths price schedule whose costs represent the maximum authorized cost.

We made no provision for any costs associated with PrEP toxicity, since most of the PrEP trials did not report any differences in the rate of serious adverse events between the study and control groups [2, 3, 12–14]. Furthermore, no severe adverse events were reported from a safety and tolerability study [15].

Generic forms of PrEP pharmaceuticals have recently become available, but from experience with other pharmaceuticals, we only expect that there will be a further 10% fall in the current maximum price authorized by the MOH. We used this 10% decrease as our baseline scenario. In our sensitivity analyses we explored scenarios where the price decrease might decrease further as a result of each HMO negotiatiating separately with the pharmaceutical suppliers.

### Treatment costs

Treatment costs were based on the global annual sum of $26,359 USD per HIV/AIDS person that is currently paid to the Israeli HMOs to cover all out-of-hospital costs incurred by their members with HIV/AIDS (Dr. Daniel Chemtob, personal communication, 2018).

In addition, monitoring costs were added based on the Ministry of Health protocols and price lists for the 81.8% of HIV-positive patients who are Israeli citizens, living in Israel in 2016.

Costs of AIDS were based on the discounted ART and monitoring costs applied to the respective survival rates of the estimated 96.5% (ie: 65% plus an estimated 90% of the original 35% who did not originally receive ART) who will receive ART and the 3.5% who did not take ART. HIV-positive persons used 4.26 general hospital days annually (Personal Communication. Ziona Haklaii, Ministry of Health Statistical Department), compared with 0.62 days use by HIV-negative persons. These 3.64 additional general hospital days a year used by HIV-positive persons were costed at an average cost of 617 USD per day [16].

However, due to lack of data on differential utilisation (by HIV-positives and negatives) of pharmaceuticals, ambulatory, emergency room and out-patient visits for diagnoses not relating to HIV, we were unable to estimate increased utilisation costs on account of HIV-positivity. No hospice costs were included as this care modality is nowadays not used any more.

### Disability weights (quality of life)

No reductions in quality of life were assumed on account of taking PrEP since clinical trials have indicated minimal side effects [2, 17]. Age-specific health utilities were multiplied by the following utilities in order to calculate utilities (or disability weights) for the following health or disease states: 1.00 for non-symptomatic HIV, 0.87 for HIV-positive taking ART [18], 0.80 for HIV-positive not taking ART [18], 0.85 AIDS case taking ART [17, 19], 0.71 AIDS case not taking ART [20, 21]. Resultant DALYs were discounted at 3% per annum.

### Epidemiology

#### Treatment impact according to different periods

From the period 2010–2013 to the period 2014–2017, there was a decrease in HIV incidence among MSM in Israel from 2.16 to 1.74 per 1000 MSM (1, MOH Department of TB and AIDS), resulting in an annual decrease of 5.20%.

This was caused by lower transmission probabilities as a result of the gradual adoption of improved preventive strategies and of improved ART drugs and protocols which lowered the viral load thresholds prescribing ART.

Due to this decline, our baseline model assumed that in the event that PrEP would not be made available (i.e. the non-intervention scenario), HIV incidence would continue to decrease at a rate of 5.20% per annum for a further four years and then remain constant. Two additional sensitivitity analyses were carried out under assumptions that there will be no further decrease and that the decrease will last for eight years more.

### Natural history of HIV and the length of time before reaching AIDS or death

In building our model, we had to consider the natural history of HIV/AIDS in order to estimate the length of time between two different clinical phases- “asymptomatic” (HIV) and symptomatic (AIDS). By definition, the natural history time estimates have to be based on a period when no HIV treatment was available.

The median time for asymptomatic HIV to “progress” to symptomatic HIV is around ten years [22]. Transition and mortality rates were based on sources both from actual trials and from modelling studies based on infectivity and frequency of sexual relations.

Relative annual age-specific progression rates from HIV to AIDS for persons not taking ART [23, 24] were applied to non age-specific Israeli data from 1981 to 96 which showed the average progression time to be 15.5 years [1]. Progression rates in persons taking ART were assumed to be 23.6% those of persons not taking ART [23–25].

Similarly relative annual age-specific mortality rates from AIDS to death for persons not taking ART [23, 24, 26, 27] were applied to (non age-specific) Israeli mortality data from 1981 to 96 which reported an average time of 5.1 years until death without treatment [1]. Mortality rates in persons taking ART were assumed to be 11.5% of persons not taking ART [23, 24, 28].

Annual age-specific mortality rates in HIV-positive persons (without an AIDS diagnosis) were calculated, assuming the rates to be 11.1 and 16.7% of those for AIDS diagnosed patients who did [23, 26, 27] and did not receive ART respectively [23, 28, 29]. Finally, an adjustment was made for gender-specific mortality rates for diagnoses not attributable to HIV or AIDS [23].

### PrEP efficacy

The essential drivers of the model included PrEP efficacy as obtained from the literature [2–5] and optimal expected compliance rates described in the Israel Ministry of Health’s PrEP evaluation proposal for the continuous regimen, and from the literature for the “on-demand” regimen.

The continuous PrEP regimen requires daily adherence to a fixed drug combination of *Tenofovir Dixoproxil Fumarate with Emtricitabine* (TDF/FTC). Users take one tablet once every 24 h [5]. The on-demand regimen includes the same fixed-dose pill (TDF/FTC), and the user is instructed to take a “loading dose” of two pills 2–24 h before sex, an additional pill 24 h after the first dose, and a final pill 48 h after the first dose. In instances of multiple consecutive intercourse, users are instructed to continue one pill every 24 h during the period, plus an additional two days [5].

Our definition of a high-risk group targetted for PrEP, were MSM who engage in unprotected anal intercourse (UAI), who account for around 21.3% of MSM [9].

Lack of accurate data precluded us from building a model based on the product of the risk of transmission, which is influenced by UAI, circumcision [30], sexually transmitted disease status [31], age-specific frequency of sexual contacts, and the probability of being an HIV carrier.

Instead, we used the following formulae to calculate (by aggregating over each age group) the expected number of incident HIV cases in MSM:

$$ \mathrm{Number}\ \mathrm{of}\ \mathrm{HIV}\ \mathrm{incident}\ \mathrm{cases}=\mathrm{Incidence}\ \mathrm{Rate}\times \mathrm{Number}\ \mathrm{of}\ \mathrm{Susceptibles} $$

$$ \mathrm{Number}\ \mathrm{of}\ \mathrm{susceptibles}=\mathrm{Number}\ \mathrm{of}\ \mathrm{MSM}-\mathrm{Number}\ \mathrm{of}\ \mathrm{MSM}\ \mathrm{HIV}-\mathrm{positive} $$

where the incidence rate for the period 2014–2017 was 174.2 per 100,000. Based on the HIV prevalence rate of 2.4% amongst MSM in Israel (MOH Department of TB and AIDS), the incidence rate among susceptibles is178.4 per 100,000.

Next, we assumed that being high-risk increases the relative risk (RR) of HIV approximately fivefold since condom use reduces heterosexual HIV transmission by 80% [32, 33]. This enabled us to calculate the number of incident cases that would occur in the high-risk and low risk (i.e.: practicing protected anal intercourse) MSM populations.

We then applied the protective efficacy of PrEP of 86% found in the clinical trials [4, 5] to estimate the reduction in HIV cases that would occur if PrEP were offered to the MSM high risk group, under the assumption that 80% of the high risk MSMs in Israel would take the opportunity to try PrEP.

In addition, we assumed in the baseline scenario that 25% of the low risk MSMs would be interested in taking PrEP, and subsequently around 75% of this group would cease to use condoms (ie: 6.25% of the low risk group would transition to high risk) [34, 35].

### Indirect costs

Our model asumed there were no differences in employment rates between general male and MSM populations, based on a Canadian study [36]. Based on the average of 40.5 h during a five-day work week for Israeli males [37] we estimated time off work to visit medical services to be 8.06 h per emergency room visit, 4.03 h for all other visits [38, 39] and 5.78 h per hospitalized day (this takes into account persons who are in hospital over weekends). Visiting frequencies were based on the Ministry of Health care protocols for ART and non-ART patients (taking into account initial confirmatory visits). Total age-specific work losses were estimated by the product of: frequency of visits, time off work per visit, the average employment cost per hour of 24.20 USD [39], and the male labour force participation rate adjusted by the unemployment rate [39]. Further indirect costs were added to take into account premature burial costs which are defined as the discounted value of burial costs of the person dying from AIDS less the discounted burial costs of dying in the future from causes other than AIDS.

**Fig. 1:**
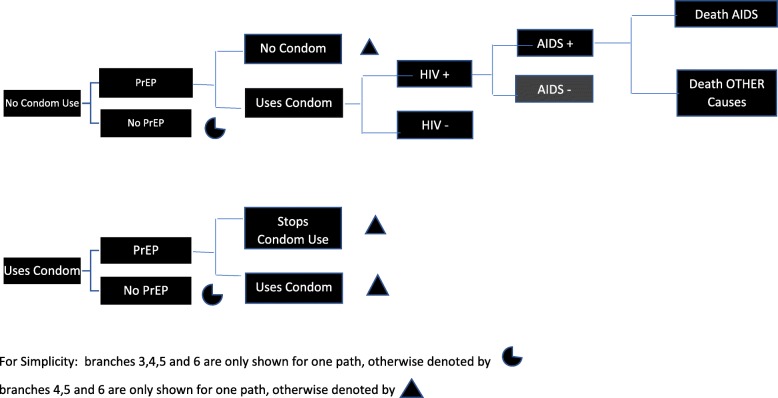
Model Structure

Figure 1 illustrates the structure of the model where each path has associated specific probabilities leading to differential path -specific outcomes, costs and utilities.

### Sensitivity analyses

These were carried out in combinations of the following scenarios:

i. The major sensitivity analysis was based on examining a range of 0–25%-50–75% (baseline) for the percentage of low risk MSM who on receiving PrEP abandon using condoms due to their enhanced feelings of protection against HIV.
ii. Assuming absolutely no low-risk MSM take up PrEP.
iii. Various efficacies of PrEP ranging from the lower 90%/95% bound from trials 56.3% [2, 4, 5] to reported efficacy of 97.1% from an observational study [10].
iv. Based on the 10% (baseline), and possible 50% & 90% [40] future declines in current pharmaceutical prices of PrEP.
v. Scenarios of zero, four (baseline) and eight years decrease in HIV incidence rates in a non-intervention scenario.
vi. The effect of altering the efficacy of PrEP was also examined for the baseline scenario..
vii. A lower cost “on-demand” regimen, assuming a 40% decrease in PrEP utilization without loss of efficacy [28, 29].
viii. The effect of adding an arbitrary average friction cost of 3000 USD representing costs to cover instances where persons have to be retrained to replace the deceased person and their specific employment skill set.
ix. Using real resource annual costs of monitoring PrEP (503 USD for first year, then subsequently 365 USD) instead of the costs based on the maximal MOH pricing lists (1179 USD for first year then subsequently 1018 USD).
x. Since some of the high-risk MSM who used PrEP, especially those who are discovered to be HIV-positive during the screening process, might start to use condoms in order to reduce their HIV transmission risk due to their greater awareness of its benefits [2, 10] we ran a sensitivity analysis that assumed between 10 and 25% high risk receivers of PrEP started using condoms as extra protection.

While we recognize that in examining the feasibility of government-funded PrEP, there is a need to consider the benefits and costs that consequently fall on other ministries or private individuals, this cost-utility calculation was based only on a health services perspective for the purpose of the sensitivity analysis.

### Decision rules

In the absence of Israel specific cost-effectiveness guidelines, decision rules were based on the WHO criteria that take into account the resources available for investment in health services in a country [41]. The PrEP intervention will be defined as being very cost-effective and cost-effective if the cost per averted DALY is less than the 40,439 USD per capita GDP of Israel [42–44] or between 1 and 3 times the per capita GDP respectively (40,439–121,316 USD). If the cost per averted DALY is more than three times the GDP (121,316USD) per capita then the intervention will be regarded as not being cost-effective. In the event that treatment is effective and treatment savings exceed intervention costs then the PrEP intervention will be cost-saving, a win-win situation [45].

As a form of sensitivity analysis we also considered a second novel stricter decision rule [46] with a cost-effectiveness threshold range of (19,463–22,208 USD at 2013 prices updated to 2018) that takes into account the opportunity costs (of the effect on health) involved in resource allocation decisions.

## Results

### Baseline case

Annual costs of providing PrEP (including monitoring costs) to the 12,666 high-risk and 14,631 low risk MSM are 188 million USD of which 15.7 and 4.9% are attributable to monitoring costs and work losses incurred by visiting clinics for monitoring.

Based on the HIV incidence rate of 178.4/100,000 susceptible MSMs, a RR of 5.0 (ie: a five-fold relative risk) for high-risk MSM [32, 33] who constituted around 21.3% of Israel’s 78,013 MSM in 2018, we estimated the annual incidences rate for both high-risk and low risk MSMs to be 421.8/100,000 and 84.4/100,000 respectively. This results in there being 65.2 new cases in the high-risk group annually and 48.2 new cases annually in the remaining low-risk group, who will not be targeted for PrEP use.

The discounted lifetime treatment, work loss and premature burial costs of an HIV-positive person (including the costs of those who regress to AIDS) are 586,436 USD, 180,363 USD and 4899 USD respectively.

Total discounted lifetime costs from the 113.4 annual incident HIV cases, amount to 75.6 million USD NIS, of which 76.0, 23.4 and 0.6% are on account of treatment costs, lost productivity and premature burials respectively.

Instituting PrEP prophylaxis over the next decade in the baseline scenario, will reduce the number of new HIV cases by 44.3% from 1113 to 621 cases, consequently saving 254, 78 and 2.1 million NIS in treatment, lost productivity and burial costs respectively. Since the intervention cost is 1898 million USD, the new net cost of the intervention (ie: intervention cost less savings) is 1564 million USD. (Table 1).

**Table 1:** ICER (USD at 2018 price levels) (Assuming 25% of non-UAI get PrEP of whom 75% of stop using condoms)

	Without PrEP	With PrEP	Change
***Assuming four years background decrease in HIV incidence of 5.2%***			
HIV cases	1113	621	−493
DALY losses	3651	2035	−1616
			Extra Costs or Savings (−)
**Costs**			
Intervention	–	1,897,994,185	1,897,994,185
Treatment Costs	574,205,146	320,086,559	−254,118,587
Productivity Losses	176,601,307	98,445,173	−78,156,144
Premature Burial	4,796,748	2,673,913	−2,122,835
TOTAL	755,603,201	2,319,199,820	1,563,596,619
Incremental Cost-Effectiveness Ratio (USD per DALY)			**967,744**
***Assuming eight years background decrease in HIV of 5.2%***			
HIV Cases	1037	578	− 459
DALY losses	3422	1908	− 1515
			Extra Costs or Savings (−)
Total Costs	708,287,160	2,292,823,802	1,583,536,642
Incremental Cost-Effectiveness Ratio (USD per DALY)			**1,046,219**
***Assuming no further background decrease in HIV***			
HIV Cases	1308	729	− 579
DALY losses	4264	2377	− 1887
			Extra Costs or Savings (−)
Total Costs	882,406,479	2,389,885,374	1,507,478,895
Incremental Cost-Effectiveness Ratio (USD per DALY)			**798,876**

Note: Assumes 80% PrEP take up by high-risk MSM,

25% PrEP take up by low-risk MSM of whom 75% stop condom use

PrEP efficacy of 86.0% (molina, mccormack)

Based on MOH payment schedule to HMOs for HIV/AIDS patients

A 10% discount on current PrEP prices

On average, each person who remains HIV-negative can expect to have a discounted healthy adjusted life expectancy (HALE) of 21.0 years, however someone who contracts HIV infection will only have a discounted HALE of 10.8 years, of which about 42% of this HALE loss is attributable to the onset of AIDS.

The introduction of PrEP in the baseline scenario (where the background decrease in HIV incidence is assumed to last for a further four years), over a decade will reduce the discounted DALYs attributable to HIV/AIDS by 1616 DALYs from 3651 to 2035 DALYs. Thus PrEP has an incremental cost (1564 million USD) utility (1616 averted DALYs) ratio of 967,744 USD per averted DALY (Table 1). As this exceeds the thrice GDP per capita guideline, the intervention is deemed to be not cost-effective. If the background decrease in HIV incidence is ignored or extended to last eight years instead of four years in the baseline case, the resultant cost-utility ratios of 798,876 USD and 1,046,219 USD per averted DALY are also both clearly far from being cost-effective (Table 1). If actual annual real resource costs of PrEP monitoring are used instead of the MOH costs used in the baseline scenario, then the cost per DALY ratio reduces only slightly to 856,769 USD per DALY.

A sensitivity analysis based on the lower 95%/90% bound of 56.3% efficacy [2, 4, 5], calculated the cost per averted DALY to be 2,689,603 USD, which fell to 754,449 USD when a 97.1% efficacy rate from an open label trial [10] was used (Table 2). Even with this higher efficacy rate, the intervention would not be cost-effective.

**Table 2:** Cost (USD at 2018 levels) per QALY by drug price and PrEP Efficacy

PrEP Efficacy						
Price	(a)	(b)	(c)	(d)	(e)	(f)
Decrease	56.3%	70.0%	78.5%	86.0%	90.0%	97.1%
10%	2,689,603	1,521,835	1,174,811	967,744	881,831	754,449
25%	2,301,819	1,290,388	989,823	810,477	736,066	625,738
50%	1,655,511	904,643	681,509	548,366	493,124	411,218
70%	1,138,465	596,047	434,858	338,677	298,771	239,603
90%	621,419	287,451	188,206	128,988	104,417	67,988

(a) Current baseline estimate

(b) lower 90%/95% bounds from refs [2, 4, 5]

(c) weighted average from refs [2, 4, 5]

(d) weighted average from refs [4, 5] (baseline)

(e) upper 90%/95% bounds from [2, 4, 5]

(f) ref. [10]

Assumes background decline in HIV for four more years, 80% PrEP take up by high-risk MSM,

25% PrEP take up by low-risk MSM of whom 75% stop condom use

Based on MOH payment schedule to HMOs for HIV/AIDS patients

Table 3 shows how the cost-utility ratios vary with changes in PrEP prices and the percentages of former non-UAI who abandon condom use on receiving PrEP. Even in the unlikely case where no-one stops using condoms, PrEP prices would have to fall by 87.5.% for cost-effectiveness to be attained. With 25, 50 and 75% decreases in condom use, cost-effectiveness would be attained if PrEP prices would fall by 88.6%, 89.6 and 90.7% respectively.

**Table 3:** Cost (USD at 2018 levels) -per averted DALY by decrease in PrEP prices and % of non-UAI users stopping Condom use on receiving PrEP

% non-UAI stopping condom use	Decrease in PrEP prices				BEP price decrease
	10%	50%	70%	90%	
0%	860,319	479,292	288,779	98,265	87.5%
25%	893,876	500,869	304,366	107,862	88.6%
50%	929,611	523,847	320,964	118,082	89.6%
75%	967,744	548,366	338,677	128,987	90.7%

Notes: Assumes 80% PrEP take up by high-risk MSM,

25% PrEP take up by low-risk MSM

PrEP efficacy of 86.0% (References (4, 5)

Based on MOH payment schedule to HMOs for HIV/AIDS patients

*BEP* Break Even Point (price decrease) for cost-effectiveness

The results were insensitive to the use of an assumed 3000 USD frictional cost to replace each deceased worker. Cost-effectiveness would be attained if prices decreased by only 88.2% compared with 90.7% if there were zero frictional costs.

Appendix 2 shows that if 10% of high risk MSM who receive PrEP take up condom use, the effect will be only to decrease the cost-utility ratios by around 1.3%. Even if one-quarter take up condom use, the effect on the cost-utility ratios will only be around a 3% decrease.

**Fig. 2:**
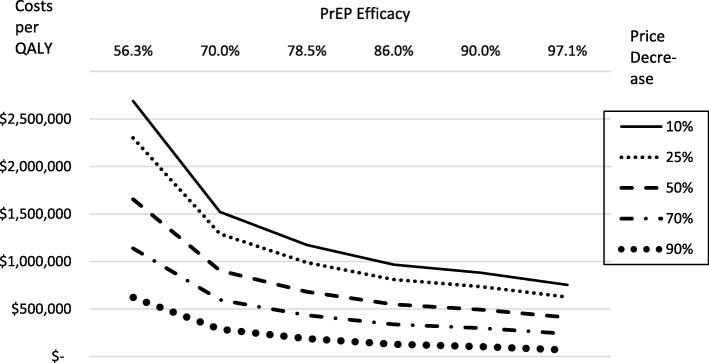
Cost (NIS) per QALY by price decrease and PrEP efficacy

The relationship between the cost-utility ratio, price decreases and PrEP efficacy is illustrated in Fig. 2 for the baseline scenario where 25% of low-risk MSMs take up PrEP and 75% stop using condoms on receiving PreP. No combination of PrEP efficacy and price decreases turned out to be cost-effective.

There could be an unlikely scenario where no low risk MSM receives PrEP, which would denote strict compliance with the proposed medical indication for PrEP use. In this scenario, supplying PrEP is cost-saving if PrEP prices fall by more than 84.0%. The intervention becomes very cost-effective and cost-effective if PrEP prices were to fall by more than 76.5%, or 61.7% respectively (Table 4).

**Table 4:** Cost-effectiveness ratios by decreases in PrEP costs assuming no non-UAI receive PrEP

(based on 2018 price levels).			
Decrease in	Net Intervention	Averted	Cost (USD) per
PrEP prices	Cost (millions USD)	DALYs	averted DALY
10%	582	1445	402,669
50%	267	1445	185,026
70%	110	1445	76,204
90%	−47(a)	1445	n.a.

Notes: Assumes 80% PrEP take up by high-risk MSM,

PrEP efficacy of 86.0% [4, 5]

(a) Negative net intervention cost means intervention becomes Cost-Saving (i.e. savings in treatment costs outweigh intervention costs) at price decreases in excess of 84.0%

Intervention becomes very cost-effective or cost-effective when PrEP prices fall by more than 76.5% or 61.7% respectively

Assuming the same efficacy could be obtained if PrEP were taken on demand as in the continuous regimen, the resultant decrease in PrEP pharmaceutical costs resulted in the baseline scenario cost-utility ratio falling to a not cost-effective 475,674 USD. Even if there were no changes in condom use, the 411,694 USD cost-utility ratio is still not cost-effective.

Clearly the use of the novel more stricter decision rule [46], only serves to emphasise the lack of cost-effectiveness of providing PrEP in our analysis.

## Discussion

Offering PrEP to all high risk MSM would have a ten year net cost of around 1564 million USD and prevent 493 persons from becoming HIV-positive, averting around 1616 DALYs. The cost per averted DALY of this intervention will be around 967,744 USD, rendering the intervention to be not cost-effective. PrEP prices would have to fall dramatically by 90.7% for the intervention to become cost-effective.

Both continuous and on-demand PrEP interventions, ranged from cost-saving to largely cost-effective in Canada [36] and were found to be cost-effective in the Netherlands [27]. Other studies reported PrEP could be very cost-effective in the USA [19, 20] especially when adherence is high. PrEP was found to be cost-effective in the USA [16] and Peru [47] and in Australia in discordant regular partnerships [48]. Cost-savings (ie: savings due to decreases in treatment costs outweigh the intervention costs) were reported from the UK [40, 49].

Our finding, of the intervention not being cost-effective is in direct contrast to what was estimated for other developed countries, as reported in the previous paragraph.

This contrast, can only partially be partially explained by the significantly lower HIV incidence rates in Israel relative to Europe and the USA. In the unlikely event that no low risk MSM used PrEP (ie: the low incidence rate is the major differential factor), we found that cost-effectiveness would indeed be achieved if PrEP prices were to fall overall by at least 61.7%. However, in reality, there are two major additional factors that differentiate cost-effectiveness in our study. Firstly, our baseline model assumed that 25% of low risk MSMs would obtain PrEP. Supplying PrEP to low risk MSM will clearly be less cost-effective than supplying PrEP to high risk MSM. Secondly, we estimated that 75% of these former low risk MSM, on receiving PrEP would cease using condoms. In effect, offsetting the protectiveness of PrEP with a higher risk behavior and requiring prices to fall by 88.6% to achieve cost-effectiveness.

Our results however, were similar to a USA modelling study that reported PrEP to be not cost-effective [41]. This could be possibly due to the fact that the USA study targetted all MSMs (i.e. low-risk and high-risk persons) as opposed to just high-risk MSM. Similarly, our study also included provision for a realistic scenario, where PrEP would also be given to some low-risk MSM, many of whom would subsequently abandon condom use.

A major disadvantage of our model is that it is static, because lack of available data (as to frequency and type of liaisons between different at-risk groups) precluded us from using a preferred transmission dynamic model**.**

Similarly, data constraints stopped us from trying to replicate the totally comprehensive models that have been reported [21, 39]. However our use of HIV incidence data enabled us to sidestep the fact that some of the behavioural individual data information were not available.

Our Israeli analysis baseline assumption of a 75% decrease in condom use by former low-risk persons who receive PrEP stands in contrast to the far lower decrease in condom use found in some trials [32, 33] and the findings of increases in condom use found in other trials [2, 10, 50]. One could hypothesise that within the framework of clinical trials more specific instructions will be given vis-à-vis the importance of condom use than those given where (as in Israel) PrEP is currently provided within the framework of the regular routine health services.

The cost-utility ratio is independent of the actual number of high-risk MSM who take up PrEP, whether due to changes in MSM prevalence or deviations from the assumed full compliance of those high-risk MSM that take up PrEP, since similar proportional decreases will occur in both net costs and averted DALYS.

Our model tended to overestimate the Cost per DALY because:-

i. lower costs would be incurred in an on-demand regimen, without any evidence that efficacy would be lower [2, 4, 5, 10].
ii. the PrEP program de facto incorporates an HIV screening programme, which has been shown to be cost-effective or even cost-saving in high risk populations [51].
iii. of the possibility that possible decreased condom use in non-UAI who receive PrEP [50] might be less than our baseline (or sensitivity analysis) figures.
iv. the underlying study [25] on which we based our symptomatic HIV to AIDS transition time, contained 22% females, who were found to have longer transition periods than males [52].
v. we were unable to estimate the increased costs for non-HIV diagnosis related pharmaceuticals, ambulatory, emergency room and outpatient visits that were likely to be incurred by HIV-positive persons.

On the other hand, the cost per DALY was possibly underestimated because:-

i. we made no provision for possible PrEP toxicity since most of the PrEP trials did not report any differences in the rate of serious adverse events between the study and control groups [2, 3, 12–14].
ii. the intervention might actuall result in decreased condom use [50] as people feel safer when they are taking PrEP [53, 54] greater than our baseline (or sensitivity analysis) figures.
iii. due to decreased condom use, the transmission of sexually transmitted infections will increase [20] which in turn increases treatment costs and decreases quality of life.
iv. we assumed there were no severe adverse events based on the results of a safety and tolerability study [15]. If however, significant toxicity does exist then the cost per QALY would be underestimated.
v. we did not include any effects and costs due to resistance caused by the use of PrEP [55]

There are other more sophisticated tools to define “high-risk” MSM than the one we used in our model. Use of these tools to estimate the risk of HIV [29] might enable us to identify additional groups of MSM that could be provided very cost-effective or even cost-saving PrEP prophylaxis.

A further cost-utility analysis could be warranted to evaluate a possible future development of providing long-acting injectable pre-exposure prophylaxis to improve compliancy with PrEP [56].

Even if a further drop in PrEP prices occurs due to generic pricing, it is apparent that the provision of PrEP to high risk MSM is not likely to be cost-effective. This is not because PrEP is ineffective, but due to a combination of relatively low incidence and high cost of the drugs, together with the high probability that many low-risk MSM may well begin taking PrEP and concurrently some of these persons will abandon condom use.

The chance of PrEP attaining cost-effectiveness would be improved if we could put in place administrative proceedures of reducing the phenomena of low-risk MSM getting PrEP and/or educational programs to reduce the phenomena of low-risk MSM who after receiving PrEP abandon condom use.

## Conclusion

Despite PrEPs high effectiveness against HIV, PrEP was found not to be cost-effective in the Israeli context because of a combination of relatively low HIV incidence, high PrEP costs, with a likelyhood that some low-risk MSM (ie: who use condoms) may well begin taking PrEP and as a consequence many of these will abandon condom use. Therefore, ways of minimizing these last two phenomena need to be found.

## Postscript

In January 2020, utilising an earlier draft of this article, the Israeli MOH negotiated with the pharmaceutical manufacturers to introduce PrEP into the National Basket of Health Services at the greatly discounted price that would achieve cost-effectiveness as estimated by the underlying model of this article.

## Data Availability

The datasets used and/or analysed during the current study are available from the corresponding author on reasonable request.

## Appendix 1

**Table 5:** Variables used in model

			Source(s) and numbered text References
**Intervention Costs**			
PrEP daily first year	24,143	NIS	[57], b
PrEP daily subsequent years	23,565	NIS	[57], b
Current discount on PrEP cost	10%		Assumed
Reduction for “PrEP on demand”	40.1%	NIS	[28, 29]
Monitoring PrEP first year	4237	NIS	a,b
Monitoring PrEP subsequent years	3659	NIS	a,b
Monitoring PrEP first year	1810	NIS	b
Monitoring PrEP subsequent years	1312	NIS	b
Employment Losses - first year	1505	NIS	[37, 39], a
Employment Losses - subsequent years	1120	NIS	[37, 39],a
**Treatment Costs**			
HIV Treatment Costs	94,759	NIS	d
monitoring NON_HAART -first year	5693	NIS	a
monitoring-NON HAART- subsequent years	12,845	NIS	a
**Disability Weights**			
HIV negative (at age 36 years)	0.09		[39, 58, 59]
HIV positive with HAART Rx	0.14		[18]
HIV no HAART Rx	0.20		[18]
AIDS with HAART Rx	0.15		[17, 19]
AIDS no HAART Rx	0.29		[20, 21]
**Epidemiology**			
Excess Hospitalization Days per HIV case	1.74	per year	[39], e
HIV incidence rate trend among MSM (2011–2017)	−5.20%	per year	f
HIV to AIDS progression with HAART	0.08	per year	[23, 24]
HIV to AIDS progression without HAART	0.33	per year	[23, 24]
HAART efficacy in Israel	47.8%		1
% HIV+ taking HAART	81.8%		f
% HIV+ of high risk group who will take HAART	81.8%		f
% of persons who were not taking HAART who start taking when they get AIDS	90%		f
***Mortality Rates (%) per year***			
HIV+ with ART- 40 yrs	0.007		[23, 26, 27]
HIV+ with ART- 50 yrs	0.013		[23, 26, 27]
HIV+ without ART- 40 yrs	0.040		[23, 28, 29]
HIV+ without ART- 50 yrs	0.073		[23, 28, 29]
AIDS+ with ART- 40 yrs	0.060		[23, 24, 26, 27]
AIDS+ with ART- 50 yrs	0.067		[23, 24, 26, 27]
AIDS+ without ART- 40 yrs	0.524		[23, 24, 28]
AIDS+ without ART- 50 yrs	0.587		[23, 24, 28]
***PreP efficacy***			
Baseline RCT	86.0%		[4, 5]
sensitivity analysis	78.5%		[2, 4, 5]
sensitivity lower 90%	56.3%		[2, 4, 5]
sensitivity upper 90%	90.0%		[2, 4, 5]
open label trial	97.1%		[10]
Coverage	100%		Assumed
% of UAI MSM starting PrEP	80%		[47]
% of non-UAI MSM starting PrEP	25%		Assumed
% ofnon-UAI MSM PrEP who cease condom use	75%		Assumption
Protection from condom use	80%		[32, 33]
Transmission risk with condom	1.6%	per act	[32, 33]
Transmission risk without condom	8.0%	per act	[32, 33]
HIV cases per 100,000 high risk MSM	421.8		f
HIV cases per 100,000 non-high risk MSM	84.4		f
**Demographic**			
Average age at HIV diagnosis	36.0	Years	f
Life expectancy at age of HIV diagnosis.	45.6	Years	[39]
Male population aged 18–69 (2018)	2,478,600		[39]
MSM prevalence	3%		[8]
MSM aged 18–69	78,013		[8, 39]
Background Mortality - age-related eg: 40 yrs	0.001064		[39]
Background Mortality - age-related eg: 50 yrs	0.002894		[39]
**Economic**			
Discount Rate	3%	per annum	[60]
Average Employment Cost	165,592	NIS per annum	[37, 39]
Exchange Rate	3.595	USD to NIS	[42]
% makes 18–69 unemployed	7.1%		[39]
Work losses due to PrEP monitoring yr 1	1505		[39]
Work losses due to PrEP monitoring yr 2+	1120		[39]
Productivity losses due to HIV	23%		[38]
% participating in labour force (age-related)	67–77%		[37, 39]
Burial Costs	30,000	NIS	Current cost
General Hospital	2218	NIS per day	b
Emergency Room Visit	316	NIS	b
% work losses due to HIV aged 15–24	23.0%		[38]
% work losses due to HIV aged 25–53	32.2%		[38]
% work losses due to HIV aged 54+	4.6%		[38]
Absenteeism due to HIV treatment	23.3	hours per year	[38], e
Social Overheads on wages	23%	NIS	[38], e
Wage Cost per Hour	71.31		[39]
Male working hours	40.475	hours per week	[37]
GNP per capita	145,374	NIS	[39, 41, 43]

a) Ministry of Health protocols

b) Ministry of Health prices

c) HMO prices

d) MOH reimbursement agreement with HMOs

e) MOH Statistics Department

f) MOH Department of TB and AIDS

## Appendix 2

**Table 6:** Cost-per averted DALY (USD) by Decrease in PrEP prices and % of non-UAI users stopping Condom use on receiving PrEP

% non-UAI stopping condom use		Decrease in PrEP prices			BEP price decrease
	10%	50%	70%	90%	
0%	849,172	472,124	283,601	95,077	87.2%
25%	882,020	493,246	298,859	104,471	88.2%
50%	916,977	515,723	315,096	114,469	89.3%
75%	954,254	539,692	332,410	125,129	90.3%

Notes: Assuming 10% of high-risk users who receive PrEP also start using condoms

Assumes 80% PrEP take up by high-risk MSM,

25% PrEP take up by low-risk MSM

PrEP efficacy of 86.0% [4, 5]

Based on MOH payment schedule to HMOs for HIV/AIDS patients

BEP = Break Even Point (price decrease) for cost-effectiveness

## Abbreviations

ART: AntiRetroviral Therapy
CUR: Cost Utility Ratio
DALY: Disability Adjusted Life Year
GDP: Gross Domestic Product
HIV: Human Immunodeficiency Virus
MOH: Ministry of Health
MSM: Men who have Sex with Men
PrEP: HIV Pre Exposure Prophylaxis
TB: Tuberculosis
TDF: Tenodir Disoproxil Fumarate
UAI: Unprotected Anal Intercourse
WHO: World Health Organization
